# The molecular pathogenesis of triptolide-induced hepatotoxicity

**DOI:** 10.3389/fphar.2022.979307

**Published:** 2022-08-24

**Authors:** Yeqing Hu, Qiguo Wu, Yulin Wang, Haibo Zhang, Xueying Liu, Hua Zhou, Tao Yang

**Affiliations:** ^1^ Institute of Cardiovascular Disease, Shuguang Hospital Affiliated to Shanghai University of Traditional Chinese Medicine, Shanghai, China; ^2^ Institute of Cardiovascular Disease of Integrated Traditional Chinese Medicine and Western Medicine, Shuguang Hospital Affiliated to Shanghai University of Traditional Chinese Medicine, Shanghai, China; ^3^ Shuguang Hospital Affiliated to Shanghai University of Traditional Chinese Medicine, Branch of National Clinical Research Center for Chinese Medicine Cardiology, Shanghai, China; ^4^ Department of Pharmacy, Anqing Medical College, Anqing, China; ^5^ Shanghai Key Laboratory of Traditional Chinese Clinical Medicine, Shanghai, China

**Keywords:** triptolide, hepatotoxicity, CYP450s, oxidative stress, immunity

## Abstract

Triptolide (TP) is the major pharmacologically active ingredient and toxic component of *Tripterygium wilfordii* Hook. f. However, its clinical potential is limited by a narrow therapeutic window and multiple organ toxicity, especially hepatotoxicity. Furthermore, TP-induced hepatotoxicity shows significant inter-individual variability. Over the past few decades, research has been devoted to the study of TP-induced hepatotoxicity and its mechanism. In this review, we summarized the mechanism of TP-induced hepatotoxicity. Studies have demonstrated that TP-induced hepatotoxicity is associated with CYP450s, P-glycoprotein (P-gp), oxidative stress, excessive autophagy, apoptosis, metabolic disorders, immunity, and the gut microbiota. These new findings provide a comprehensive understanding of TP-induced hepatotoxicity and detoxification.

## 1 Introduction

*Tripterygium wilfordii* Hook. F (*Tw*HF), the dried root or root xylem of *Tripterygium wilfordii* Hook. f. *Tw*HF and its preparations, have been widely used to treat autoimmune and inflammatory diseases because of its significant anti-inflammatory and immunosuppressive effects (Luo et al., 2019). Triptolide (TP) is a diterpene triepoxide, which is the major pharmacologically active ingredient and toxic component of *Tw*HF (Xi et al., 2017). TP has attracted the attention of researchers because of its high potential for clinical application, such as its anti-inflammatory, anti-cancer, anti-immune, and anti-oxidant effects (Guo et al., 2016; Yu et al., 2021; Gao et al., 2022; Song et al., 2022). However, its clinical potential is limited by a narrow therapeutic window and multiple organ toxicity, especially hepatotoxicity (Zhou et al., 2012; Xi et al., 2017).

The data on adverse reactions (ADRs) of *Tw*HF preparations containing TP released by the National Adverse Drug Reaction Monitoring Center of China shows that 839 cases of ADRs caused by *Tw*HF preparations from 2004 to September 2011, mainly manifested as drug-induced hepatitis and renal insufficiency. Furthermore, there are 472 cases of ADRs were reported in *Tripterygium wilfordii* polyglycosides from 1 September 2014 to 31 August 2019 and 54 cases (11.44%) of which were abnormal liver function. It was reported that the distribution concentration of TP in rat liver was more than three times that of other tissues, which might be the reason for the significant hepatotoxicity among the multi-organ toxicities (Mei et al., 2012). Clinically, the hepatotoxicity caused by *Tw*HF and its preparations containing TP is mainly manifested as acute hepatitis, with symptoms such as nausea, fatigue, anorexia, increased serum alanine transaminase (ALT)and aspartate aminotransferase (AST) levels, and obvious cholestasis (Yang et al., 2018). Focal necrosis, inflammatory cell infiltration, cell degeneration, apoptosis, and bile duct hyperplasia are the common pathological manifestations in TP-induced hepatotoxicity (Wang X. Z. et al., 2018; Zou et al., 2020).

The hepatotoxicity mechanisms of TP are quite complex and show significant inter-individual variability. Researchers have explored the mechanism of TP-induced hepatotoxicity. Therefore, this review will provide a comprehensive analysis of the toxicological mechanism of TP to promote our understanding of TP-induced hepatotoxicity and detoxification.

## 2 The mechanisms of TP-induced hepatotoxicity

### 2.1 CYP450s and P-gp mediated hepatotoxicity

TP, an active electrophile containing three epoxide groups, can bind covalently to a 90-kda protein, XPB (Xeroderma pigmentosum, complementation group B), thereby inhibiting XPB’s ATPase activity, which might be the underlying reason for the toxicity of TP (Titov et al., 2011). Opening the epoxide ring of TP can reduce its toxicity (Zhang and Li, 2004). Clinically, the therapeutic dose of ,*TW*HF tablets is about 4-6 tablets/d, which is equal to 400–600 μg/d TP (Qu et al., 2015). In addition, the effective dose of TP could reach 400 μg/kg administered continuously for 40 days in the animal experiments (Pan et al., 2017). However, the acute toxicity study showed that administration of 500 μg/kg in mice could cause liver injury (Wang et al., 2016), and the acute toxicity dose converted into 60 kg of human is 2,400 μg. These data implicate a narrow therapeutic window of TP and a slight excess of TP can lead to the risk of hepatotoxicity. Furthermore, studies have demonstrated that TP-induced hepatotoxicity is dose- and time-dependent (Yuan et al., 2019a; Zhao J. et al., 2020). In addition, cytochrome P450 (CYP) enzymes are involved in TP metabolism and P-glycoprotein (P-gp) is involved in TP transport, which affects the *in vivo* processing of TP (Xue et al., 2011; Zhang et al., 2016). Therefore, the abnormal increase TP exposure caused by CYP450s and P-gp, which leads to the increase of TP’s direct hepatotoxicity, is an important factor for its hepatotoxicity.

#### 2.1.1 CYP450s

##### 2.1.1.1 Abnormal metabolism of CYP450 enzyme systems

CYP450s, a superfamily of enzymes, catalyze phase I oxidation and reduction of endogenous and xenobiotics substances (Nebert and Dalton, 2006). Studies have demonstrated that CYP450-mediated phase I metabolism of TP is the major pathway for reducing hepatotoxicity. Glutathione (GSH)-conjugated phase II metabolism induces mild detoxification and plays a minor role in TP-induced hepatotoxicity (Du et al., 2014; Li et al., 2015). Comparing liver-specific CYP450 reductase knockout mice and wild-type mice, the inactivation of hepatic CYP450 could significantly increase TP bioavailability and levels in liver tissue, leading to increased hepatotoxicity (Xue et al., 2011). Similarly, the CYP450 enzyme inhibitor 1-aminobenzotriazole or the CYP450 enzyme activator phenobarbital could increase or decrease the TP level, thereby modulating TP-induced primary rat hepatocyte toxicity (Jin et al., 2019). However, compared with wild-type mice, there were no significant differences in hepatotoxicity and pharmacokinetics in mice with low levels of extrahepatic CYP450 reductase, demonstrating that extrahepatic CYP450 has no effect on TP-induced hepatotoxicity (Wei et al., 2018). These studies confirmed the important role of hepatic CYP450 enzyme-mediated TP metabolism in TP-induced hepatotoxicity.

CYP450 isoforms make different contributions to TP metabolism and hepatotoxicity. CYP3A4 and CYP2C19 are involved in the metabolism of TP in male human liver microsomes, and CYP3A4 is the main isoform responsible for its hydroxylation (Li et al., 2008). Dexamethasone, a CYP3A inducer, increases TP metabolism and reduces TP-induced hepatotoxicity in rats (Ye et al., 2010). Another *in vitro* experiment confirmed that CYP3A-mediated TP metabolism can modulate TP-induced hepatotoxicity (Shen et al., 2014).

TP has been investigated to inhibit CYP450s. It was reported that TP exposure has a weak and time-dependent inhibitory effect on CYP3A in rat liver microsomes and hepatocytes (Shen et al., 2014). Likewise, TP inhibited the activity of human hepatic CYP1A2 and CYP3A4, but not other CYP450 isoforms, such as CYP2A6, 2E1, 2D6, 2C9, 2C19, and 2C8 (Zhang et al., 2017). Furthermore, long-term oral administration of TP could significantly inhibit the expression and activity of CYP3A4 in rats (Zhang et al., 2014). Researchers further investigated the effect of TP on the six major subtypes of CYP450 (1A2, 2C9, 2C19, 2D6, 2E1, and 3A). The result showed that continuous gavage of TP for 28 days could inhibit the activity and expression of CYP450 isoforms 3A, 2C9, 2C19, and 2E1 and cause hepatotoxicity in rats (Lu et al., 2017). In addition, TP could inhibit the transcriptional activation of the pregnane X receptor (PXR), thereby inhibiting the expression and function of metabolic detoxification enzymes, especially CYP3A4, which ultimately led to reduced metabolic elimination of TP and increased hepatotoxicity (Zheng et al., 2021). From the above studies, we speculated that the inhibitory effect of TP on CYP450s can cause alterations in TP metabolism and hepatotoxicity. However, whether TP inhibits CYP2E1 and CYP2C9 remains controversial.

Besides, studies have shown that CYP450s are also involved in endogenous substance metabolism and reactive oxygen species (ROS) production. A metabolomic study showed that TP treatment induced metabolic disorders, such as changes in carnitine, lysophosphatidylcholine (LPC), and a series of amino acids in female mice, and regulating the activity of CYP3A4 can modulate LPC levels, amino acid levels, the glutathione (GSH)/Glutathione disulfide (GSSG) ratio, and oxidative stress, thereby altering TP-induced hepatotoxicity (Xiao et al., 2020). Moreover, the TP-induced increase in CYP2E1 expression induces hepatic oxidative stress (Jiang et al., 2021). In C57 mice and IHHA-1 cell models, CYP2E1 expression was upregulated after TP exposure, which not only directly increased ROS levels, but also increased the expression of nuclear factor kappa B (NF-κB) (p65) to mediate oxidative stress and inflammatory responses (Jiang et al., 2021).

##### 2.1.1.2 Sex differences, genetic polymorphisms and circadian rhythms

A significant difference in the hepatotoxicity of TP was found between different sexes in rats. The expression of CYP3A2, which is involved in TP metabolism, is higher in male rats than in female rats, a factor that makes females more susceptible to TP-induced hepatotoxicity (Liu et al., 2010). Inhibiting the expression and activity of CYP3A2 in male neonatal rats could eliminate the sex-dependent differences in TP metabolism (Liu et al., 2011).

Synonymous nucleotide polymorphisms can affect gene expression and function. Chen et al. established stable HepG2 cells with lentiviral vectors expressing either *CYP2E1-1263C/wt* or *CYP2E1-1263T/mt* constructs to investigate the effect of synonymous SNP rs2515641 on *CYP2E1* expression, and found that both the mRNA and protein expression levels of CYP2E1 were significantly decreased, and the ALT level of CYP2E1-1263T/mt recombinant cells was increased compared with that in the CYP2E1-1263C/wt recombinant cells after TP treatment for 48 h, indicating that synonymous mutation rs2515641 induced susceptibility to TP-induced hepatotoxicity by affecting CYP2E1 mRNA and protein expression (Chen et al., 2020).

Clock proteins affect drug metabolism and drug detoxification by regulating CYP450s (Zhao et al., 2019). A pharmacokinetic experiment showed that gavage with *TwHF* at Zeitgeber time 2 (ZT2) generated higher plasma concentrations of TP and more severe liver injury compared with ZT14 dosing, which was associated with circadian expression of hepatic CYP3A11 regulated by the circadian clock (Zhao H. et al., 2020). Likewise, the core clock gene *Bmal1* (encoding brain and muscle ARNT-like 1) controls the circadian rhythm of CYP3A11 by regulating D-box binding PAR BZIP transcription factor (DBP)/hepatocyte nuclear factor 4 alpha (HNF4α), TP-induced hepatotoxicity exhibited more severe daytime (ZT2/8) than nighttime (ZT14/20) toxicity (Lin et al., 2019).

#### 2.1.2 P-gp

P-gp, an efflux transporter, attenuates intestinal absorption, limits penetration of the blood-brain barrier, and mediates biliary and urinary excretion of substrate drugs (Zamek-Gliszczynski et al., 2021). The bidirectional transport of TP across Caco-2 cells was investigated *in vitro*, which showed that the transport of TP in Caco-2 cells was significantly increased in the basolateral to the apical direction and was abolished in the presence of the P-gp inhibitor, verapamil, indicating that P-gp is involved in intestinal absorption of TP (Gong et al., 2015). A Rhodamine-123 uptake assay confirmed that TP did not affect the activity of P-gp in Caco-2 Cells (Zhang et al., 2017). Therefore, P-gp protein activity does not change when TP is used alone, and the absorption of TP might not be altered. However, when TP is combined with drugs that interfere with P-gp activity, the absorption of TP, which might lead to changes in hepatotoxicity.

TP is mainly secreted in bile (Liu et al., 2014). A recent study showed that P-gp is involved in TP clearance and detoxification in a Sandwich-cultured rat hepatocyte model, and identified TP as a P-gp substrate using a rat *Mdr1* (the gene responsible for encoding the P-gp protein) membrane ATPase assay (Zhuang et al., 2013). Small interfering RNA (siRNA) and the specific inhibitor tariquidar were used to downregulate hepatic P-gp levels, which significantly increased the plasma and hepatic exposure of TP in mice, resulting in severe hepatotoxicity (Kong et al., 2015). The above research indicates that hepatic P-gp regulates TP-induced hepatotoxicity by affecting its clearance.

### 2.2 Oxidative stress mediated hepatotoxicity

Oxidative stress (OS) is caused by an imbalance between oxidation and antioxidants. OS causes lipid and DNA damage, leading to organelle dysfunction and damage (Pole et al., 2016; Campbell and Colgan, 2019). Numerous *in vivo* and *in vitro* studies have found that administration of TP causes an increase in ROS and malondialdehyde (MDA), and a decrease in superoxide dismutase (SOD), catalase (CAT), glutathione-S-transferase (GST) and GSH (Li et al., 2014; Jiang et al., 2021). Thus, TP-induced hepatotoxicity is associated with oxidative stress.

#### 2.2.1 Mitochondrial oxidative stress

Mitochondria are important sites for biosynthetic processes that regulate stress responses (Vakifahmetoglu-Norberg et al., 2017). TP increased the mitochondrial membrane transmembrane potential (Δ*Ψ*m) in a concentration-dependent manner in HepG2 and HL7702 cells, which increased oxidative stress (Fu et al., 2011; Fu et al., 2013). In addition, TP inhibited the activity of mitochondrial respiratory chain complexes I and IV in a concentration-dependent manner, leading to obstruction of electron flow, causing the accumulation of electrons in the upstream respiratory chain complex, which reacts with oxygen through the upstream complex to form superoxide anion radical (O^2−^), thereby increasing ROS and inducing oxidative stress (Fu et al., 2011). Moreover, in mice, inhibition of the mitochondrial respiratory chain by TP induced secondary β-oxidative damage, microvesicular steatosis, increased lactate and ROS, and decreased GSH, resulting in liver injury (Fu et al., 2011). Moreover, TP caused mitochondrial dysfunction by inducing mitochondrial swelling and permeability in female rat liver mitochondria (Fu et al., 2013). Notably, in a rat model of TP-induced hepatotoxicity, TP upregulated the expression of the mitochondrial fission-related, dynamin-related protein 1 (Drp1), leading to disturbances in mitochondrial dynamics and mitochondrial dysfunction, such as increased ROS, DNA membrane depolarization, decreased ATP production, and decreased mitochondrial DNA copy number (Hasnat et al., 2019; Hasnat et al., 2020). Taken together, these studies proved the importance of mitochondrial oxidative stress in TP-induced hepatotoxicity.

#### 2.2.2 Imbalance of antioxidant

In addition to oxidative stress caused by TP-induced mitochondrial dysfunction, oxidative stress caused by TP-induced imbalance of cellular antioxidant protect systems is also an important mechanism of TP-induced hepatotoxicity.

Nuclear factor erythroid 2-related factor 2 (Nrf2) is a redox-sensitive transcription factor that regulates cellular oxidative stress (Lee et al., 2005). Upregulating *NRF2* expression can reduce TP-induced HepG2 cytotoxicity, whereas silencing *NRF2* can raised oxidative stress and aggravated triptolide-induced cytotoxicity in HepG2 and L02 cells (Li et al., 2014; Zhou et al., 2017). Also, treatment with the canonical Nrf2 agonist sulforaphane (SFN) attenuated TP-induced hepatotoxicity by reducing oxidative stress in mice (Li et al., 2014). *In vitro*, TP enhanced *NRF2* expression, induced Nrf2 nuclear translocation, increased Nrf2 adenine-uridine rich element (ARE)-binding activity, increased heme oxygenase 1 (HO-1) and NAD(P)H quinone dehydrogenase 1 (NQO1) expression in L-02 and HepG2 cells (Zhou et al., 2017). MicroRNA-155 (miR-155), an endogenous non-coding small RNA, was upregulated in a TP concentration-dependent manner, resulting in downregulation of the Nrf2 signaling pathway, and inhibition of miR-155 reversed the downregulation of the Nrf2 signaling pathway by TP and alleviated TP-induced hepatotoxicity (Li et al., 2021). Furthermore, TP treatment can inhibit the endogenous peroxisome proliferator-activated receptor alpha (PPARα) signaling pathway, leading to increased long-chain acylcarnitines in mouse serum, which in turn activated the Notch-Nrf2 pathway to protect against TP-induced hepatotoxicity (Hu et al., 2019). According to the above research, there is controversy regarding whether TP inhibits or upregulates Nrf2. What is clear, however, is that activation of Nrf2 protects can against TP-induced hepatotoxicity.

Activation of the Notch1 signaling pathway has been shown to reduce oxidative stress in injured tissues (Cai et al., 2022). The thioredoxin (TRX) system is a key antioxidant system against oxidative stress (Lu and Holmgren, 2014). A study demonstrated that TP could inhibit Notch1 expression, impaired the antioxidant activity of TRX through the phosphatase and tensin homolog (PTEN)/protein kinase B (AKT)/thioredoxin interacting protein (Txnip) signaling pathway, and caused oxidative damage (Shen et al., 2019).

Taken together, oxidative stress is involved in TP-induced hepatotoxicity, and regulation of Drp1, or activation of Nrf2 and Notch1 can alleviate oxidative stress and TP-induced hepatotoxicity.

### 2.3 Excessive autophagy-mediated hepatotoxicity

Autophagy is a lysosomal degradation pathway through which cellular contents, such as damaged organelles and proteins, are recycled (Xie and Klionsky, 2007). A study showed that TP-induced oxidative stress led to a time- and dose-dependent increase in autophagy-related proteins Beclin1 and membrane-bound microtubule-associated protein light chain 3 (LC3II). The study identified the presence of autophagosomes by transmission electron microscopy, and inhibition of autophagy decreased cell viability and enhanced apoptosis in a TP-induced HL7702 cytotoxicity model (Wei et al., 2019). This indicated that autophagy plays a protective role in TP-induced hepatotoxicity. Yet, in another *in vitro* experiment, it was shown that TP exposure led to excessive autophagy induced by endoplasmic reticulum stress (ERS) through the PERK-ATF4-CHOP pathway. Co-treatment with the autophagy agonist RAPA increased TP-induced cytotoxicity and apoptosis, while co-treatment with the autophagy inhibitor 3-MA decreased TP-induced cytotoxicity and apoptosis (Zhang et al., 2022b). This may due to the unclarified role that autophagy plays in the TP-induced hepatotoxicity. Appropriate autophagy can protect TP-induced hepatotoxicity, while excessive autophagy aggravates TP-induced hepatotoxicity.

Besides, electron microscopy showed obvious mitophagy in TP-treated rat liver tissue, and mitophagy was related to the disturbance of mitochondrial function and dynamics caused by the TP-induced increase in Drp1 (Hasnat et al., 2019). However, that study did not further verify the role of mitophagy in TP-induced hepatotoxicity. Furthermore, another study found that TP-induced ERS could induce excessive mitophagy in HepaRG cells. Co-treatment with Mdivi-1, a specific inhibitor of mitophagy could alleviate TP-induced HepaRG cytotoxicity (Zhang et al., 2022c).

Collectively, TP-induced ERS lead to excessive autophagy, and modulation of autophagy can alleviate TP-induced hepatotoxicity.

### 2.4 Apoptosis-mediated hepatotoxicity

Apoptosis is a programmed death process that is mainly triggered by two basic pathways: The death receptor pathway and the mitochondrial pathway, both of which ultimately lead to the activation of caspases (Dara and Kaplowitz, 2017; Galluzzi et al., 2018). Using transcriptomic techniques, among apoptosis-related genes, 46 genes were upregulated and 43 genes were downregulated in TP-induced hepatotoxicity in Wistar rats (Wang et al., 2013). In another transcriptomics study, TP-induced apoptosis through regulating the phosphatidylinositol-4,5-bisphosphate 3-kinase (PI3K)/AKT signaling pathway, the tumor necrosis factor alpha (TNFα) signaling pathway, the mitogen-activated protein kinase (MAPK) signaling pathway, and the P53 signaling pathway; however, these pathways have not been further validated (Zhao J. et al., 2020). These studies indicated that apoptosis is associated with TP-induced hepatotoxicity.

TP exposure caused loss of mitochondrial membrane potential and the release of cytochrome c from mitochondria into the cytoplasm, which leads to downregulation of the level of the anti-apoptotic protein, B-cell CLL/lymphoma 2 (Bcl-2), upregulation of the level of the pro-apoptotic protein BCL2 associated X (Bax) and the tumor suppressor protein P53, and an increase in the activity of caspase 9 and caspase 3, ultimately causing apoptosis in L-02 cells (Yao et al., 2008). An in-depth study showed that TP treatment induced dysfunction and altered dynamics of mitochondria by causing the translocation of Drp1 from the cytoplasm to the mitochondrial outer membrane, with concomitant release of cytochrome c from mitochondria into the cytoplasm, which activated caspase 3, leading to apoptosis (Hasnat et al., 2020). TP caused an increase in ROS by inhibiting Notch1, which ultimately induced apoptosis, and upregulating the expression of Notch1 can reduce apoptosis and alleviate TP-induced hepatotoxicity (Shen et al., 2019). In general, TP-induced mitochondrial damage and oxidative stress are important factors in triggering apoptosis.

### 2.5 Hepatotoxicity mediated by metabolic disorders

Bile duct hyperplasia and lipid accumulation were observed in the histological manifestations of TP-induced hepatotoxicity (Fu et al., 2011; Jin et al., 2015), which indicated that metabolic disorders are involved in TP-induced hepatotoxicity.

#### 2.5.1 Bile acid metabolism

The farnesoid X receptor (FXR) is a ligand-activated transcription factor that activates certain genes involved in phases II and III of xenobiotic metabolism, protecting the liver from toxic metabolites and xenobiotics (Lee et al., 2010). Pretreatment with the FXR activator, GW4046, has been shown to maintain metabolism homeostasis and attenuate TP-induced hepatotoxicity by increasing gene expression related to phase II and phase III xenobiotic metabolism (Jin et al., 2015). Besides, FXR also plays a key role in maintaining hepatic bile acid homeostasis (Molinaro and Marschall, 2022). Additionally, sirtuin 1 (Sirt1) regulates FXR activation, and depletion of hepatic Sirt1 reduces FXR signaling (García-Rodríguez et al., 2014). TP suppressed the Sirt1/FXR signaling pathway by inhibiting Sirt1 activity, which led to a decrease in the expression of the bile salt export pump (BSEP), an increase in the expression of bile acid synthesis rate-limiting enzyme CYP7A, and a decrease in the expression of gluconeogenesis proteins glucose-6-phosphatase catalytic subunit (G6PC) and phosphoenolpyruvate carboxykinase 2, mitochondrial (PEPCK), resulting in the destruction of bile acid homeostasis and gluconeogenesis, thus causing liver injury in rats (Yang et al., 2017). Zhou et al. (Zou et al., 2020) found that TP induced the activation of iNKT cells, which in turn led to decreased expression of the cholestasis-related nuclear receptor FXR, transporter organic anion-transporting polypeptide 1b2 (OATP1B2), and CYP450 enzymes, which was also responsible for aggravating cholestatic liver injury.

#### 2.5.2 Lipid metabolism

A study suggests that sex differences in TP-induced hepatotoxicity might be related to dyslipidemia. Continuous gavage of TP resulted in significant upregulation of the liver X receptor-alpha (LXR)/sterol regulatory element binding transcription factor 1 (SREBP-1) signaling axis in females, while male rats showed only slight changes, resulting in more lipid droplets in TP-treated female rats than in TP-treated male rats, which might be a factor in the sex differences in TP-induced hepatotoxicity (Jiang et al., 2016). In addition, targeted fatty acids analysis showed that TP exposure induced significant changes in fatty acid levels in the livers of rats and mice (Li et al., 2017; Liu et al., 2021).

#### 2.5.3 Other metabolisms

Moreover, several untargeted metabolomics studies showed that a variety of metabolic pathway disorders can be found in TP-induced hepatotoxicity, including glutathione metabolism, purine metabolism, and glycerophospholipid metabolism (Zhao J. et al., 2020; Yang et al., 2022). Additionally, targeted amino acid and sphingolipid analysis revealed that TP exposure induced changes in amino acid and sphingolipid metabolism profiles (Qu et al., 2015; Hu et al., 2021). However, no in-depth research has been carried out.

### 2.6 Immune-mediated hepatotoxicity

As an immune organ, the liver is enriched with a large number of innate immune cells, such as neutrophils, Kupffer cells (KCs), dendritic cells (DCs), and natural killer cells (NKs), as well as adaptive immune cells, such as T cells and B cells. Evidence demonstrates that the immune response is closely linked to the development of drug-induced liver injury (DILI) (Jiang et al., 2020; Nguyen et al., 2022).

#### 2.6.1 The innate immune system

NLR family pyrin domain containing 3 (NLRP3), a multi-protein complex assembled in response to the activation of intracellular pattern recognition receptors (PRRs), is an important component of the innate immune system. NLRP3 can be activated by damage-related molecular patterns (DAMPs) produced by damaged cells and pathogen-related molecular patterns (PAMPs) generated by enterohepatic axis pathogens (Kanneganti et al., 2007; Yu et al., 2022). Activation of the NLRP3 inflammasome releases inflammatory factors IL-1β and IL-18, which initiate an inflammatory cascade (Baroja-Mazo et al., 2014; Bruchard et al., 2015; Tsai et al., 2017). Research has shown that activation of the TLR4-MYD88 innate immune signal transduction adaptor (Myd88)-NF-κB pathway and oxidative stress by TP are involved in the overactivation of the NLRP3 inflammasome, which releases a large amount of IL-1β and increases the recruitment of neutrophils and macrophages, thereby aggravating liver injury (Yuan et al., 2019a). A single dose of Ac-Yvad-Cmk (a Caspase 1 inhibitor) injected before TP administration could effectively protect against TP-induced hepatotoxicity (Yuan et al., 2019a).

Hepatic macrophages are the most abundant hepatic immune cells which play a vital role in maintaining hepatic homeostasis and contribute to the progression of DILI (Tsuji et al., 2020). Several studies have shown that TP inhibited the phagocytosis of macrophages, increased the number of macrophages, and modulated the polarization of macrophages in mice (Wang L. et al., 2018; Liu L. et al., 2022). what’s more, in a study of TP-induced acute hepatotoxicity, researchers found that depletion of macrophages using clodronate liposomes pretreatment could suppress the inflammatory response and alleviated TP-induced hepatotoxicity (Zhao, 2018).

Neutrophils, the innate immune cells, are the most abundant leukocytes in mammals (Tang et al., 2021). It has been found that neutrophils increasingly infiltrate the liver following TP treatment because of activation of the NLRP3 inflammasome, induction of the TLR4 and TLR9 signaling pathway, release of inflammatory factors, or activation of other immune cells (Wang X.z. et al., 2018; Wang X. Z. et al., 2018; Yuan et al., 2019a). A study showed that neutrophil infiltration had little effect on TP-induced hepatotoxicity, because depletion of neutrophils only showed mild protection against TP-induced hepatotoxicity (Wang X. Z. et al., 2018).

Natural Killer T (NKT) cells express both NK and T cell surface markers, linking innate and adaptive immunity (Kaer et al., 2011). Among them, invariant NKT (iNKT) cells are a subset of cells that cause liver inflammation and play a pathogenic role (Maricic et al., 2018; Marrero et al., 2018; Nong et al., 2020). NKT, especially iNKT, cells are increasingly activated in the liver after TP administration, resulting from the upregulation of the Toll-like receptor (TLR) signaling pathway, which mainly releases the Th1 cytokine interferon gamma (IFN-γ), recruits neutrophils and macrophages, and causes hepatotoxicity (Wang X.z. et al., 2018). Further research demonstrated that TP-activated iNKT cells released significantly increased levels of the Th2 cytokine, IL-4, thereby inhibiting type 2 NKT cells and promoting the expression of C-X-C motif chemokine ligand 10 (CXCL10), intercellular adhesion molecule 1 (ICAM-1), and early growth response 1 (Egr-1), resulting in downregulated expression levels of liver nuclear receptor FXR, bile acid transporter organic anion-transporting polypeptide 2b1 (OATP2B1), and CYP450 enzymes, ultimately exacerbating TP-induced cholestatic liver injury (Zou et al., 2020).

#### 2.6.2 The adaptive immune system

The balance of T helper cell 17 (Th17)/regulatory T cell (Treg) in adaptive immune cells is crucial to maintain immune homeostasis (Liu et al., 2018; Lee et al., 2021). Tregs have anti-inflammatory effects that suppress immune responses. In contrast, Th17s have pro-inflammatory effects to promote immune responses. Increased levels of Th17s and their related cytokine IL-17, but decreased levels of Treg cells and their related cytokine IL-10, were observed in TP-induced hepatotoxicity (Wang et al., 2014a). Another study found the number of Tres was reduced by TP-mediated upregulation of suppressor of cytokine signaling (SOCS) and Notch signaling, resulting in a Th17/Treg imbalance and aggravated TP-induced hepatotoxicity (Wang et al., 2016). Furthermore, in mice, IL-17 produced by Th17s contributed to TP-induced hepatotoxicity (Wang et al., 2014b).

#### 2.6.3 Liver immune homeostasis

Mild bacterial-derived lipopolysaccharide (LPS) in the gut could enter the liver through the portal vein. Under physiological conditions, the hepatic immune system protects the host from the adverse effects of low-dose LPS, while mice that were gavaged daily with TP for 7 days showed disrupted liver immune homeostasis, evidenced by the liver’s inability to detoxify the harmful response induced by a non-hepatotoxic dose of LPS (Yuan et al., 2019b). Researchers further found that the imbalance of hepatic immune homeostasis was caused by TP-induced inhibition of NF-κB-dependent transcriptional activity and CASP8 and FADD like apoptosis regulator (CFLAR, also known as FLIP) production (Yuan et al., 2020). Furthermore, TP inhibited the phagocytic function of macrophages, increased the number of macrophages, and regulated macrophages polarization in the mouse liver, resulting in an amplification of the inflammatory response to a non-hepatotoxic dose of LPS and exacerbated hepatotoxicity (Wang L. et al., 2018; Liu L. et al., 2022). Recently, it was found that the immune disorder caused by a TP-induced Th17/Treg imbalance was also the cause of liver intolerance to a non-hepatotoxic dose of LPS in TP-induced acute hepatotoxicity (Zhang H. et al., 2022). Hence, TP treatment led to an imbalance of immune homeostasis in the liver, making the liver susceptible to external factors, such as LPS, thereby aggravating liver damage.

### 2.7 Gut microbiota mediated hepatotoxicity

The close connection between the liver and the gut, such as the “gut-liver axis” and “enterohepatic circulation” means that the gut microbiota has an important impact on the efficacy and toxicity of drugs (Kashyap et al., 2017; Wilson and Nicholson, 2017; Andrade et al., 2019). Enzymes or metabolites derived from the gut microbiota alter DILI by affecting the *in vivo* processing of drugs (Gong et al., 2018; Yip et al., 2018; Li et al., 2019). Moreover, the gut microbiota and its metabolites can regulate liver injury by influencing immunity and the gut barrier (Sun et al., 2020; Yan et al., 2022).

In recent years, researchers have focused on the association between the gut microbiota and TP-induced hepatotoxicity. An *in vitro* experiment on SD rat colon contents demonstrated that TP could be metabolized by the gut microbiota and the content of TP showed a downward trend over time, with a decrease of 3% at 6 h, 16% at 12 h, and 45% at 24 h (Peng et al., 2020). In mice, TP injection after antibiotic depletion of the gut microbiota increased TP-induced hepatotoxicity, and the mechanism was related to metabolic disorders. Gut microbiota-derived propionate was reported to defend against TP-induced hepatotoxicity through metabolic intervention (Huang et al., 2020). Considering that most TP-containing drugs are delivered orally, our study further demonstrated that gut microbiota disturbances induced by antibiotic pretreatment exacerbated oral TP-induced hepatotoxicity, which is associated with an aggravated NLRP3-mediated inflammatory response and increased TP absorption (Liu Y. T. et al., 2022).

## 3 Conclusion

**FIGURE 1:**
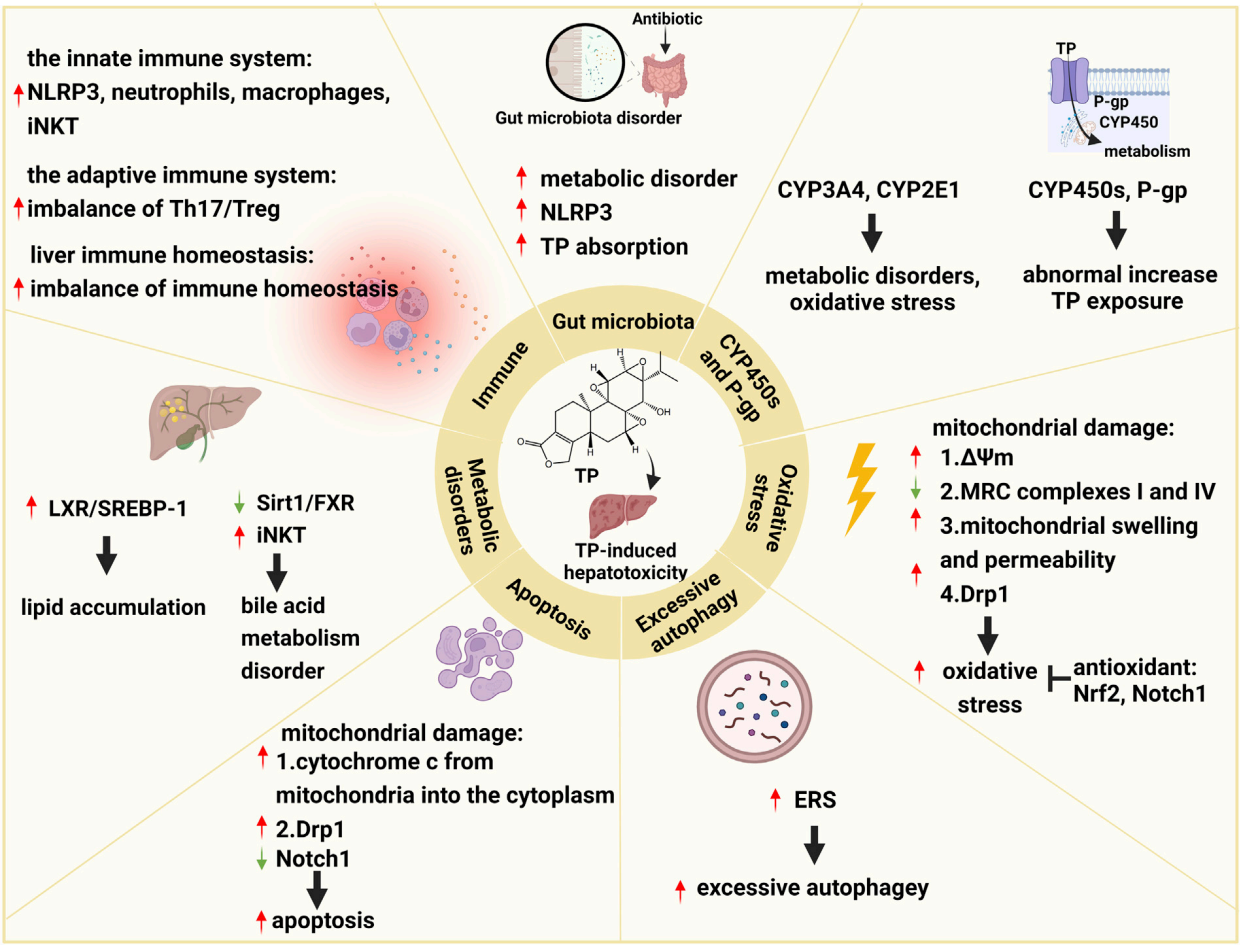
Schematic diagram of molecular pathogenesis of TP-induced hepatotoxicity. This figure shows that TP-induced hepatotoxicity is mainly related to CYP450s, P-gp, oxidative stress, excessive autophagy, apoptosis, metabolic disorders, immunity, and the gut microbiota. ↑: upregulation; ↓: downregulation; ┬: inhibition.

The pathogenesis of TP-induced hepatotoxicity is complicated, and the occurrence of TP-induced hepatotoxicity is the result of the interaction of multiple factors. In summary, the mechanisms of TP-induced hepatotoxicity are mainly related to CYP450s, P-gp, oxidative stress, excessive autophagy, apoptosis, metabolic disorders, immunity, and the gut microbiota (Figure 1).

**FIGURE 2:**
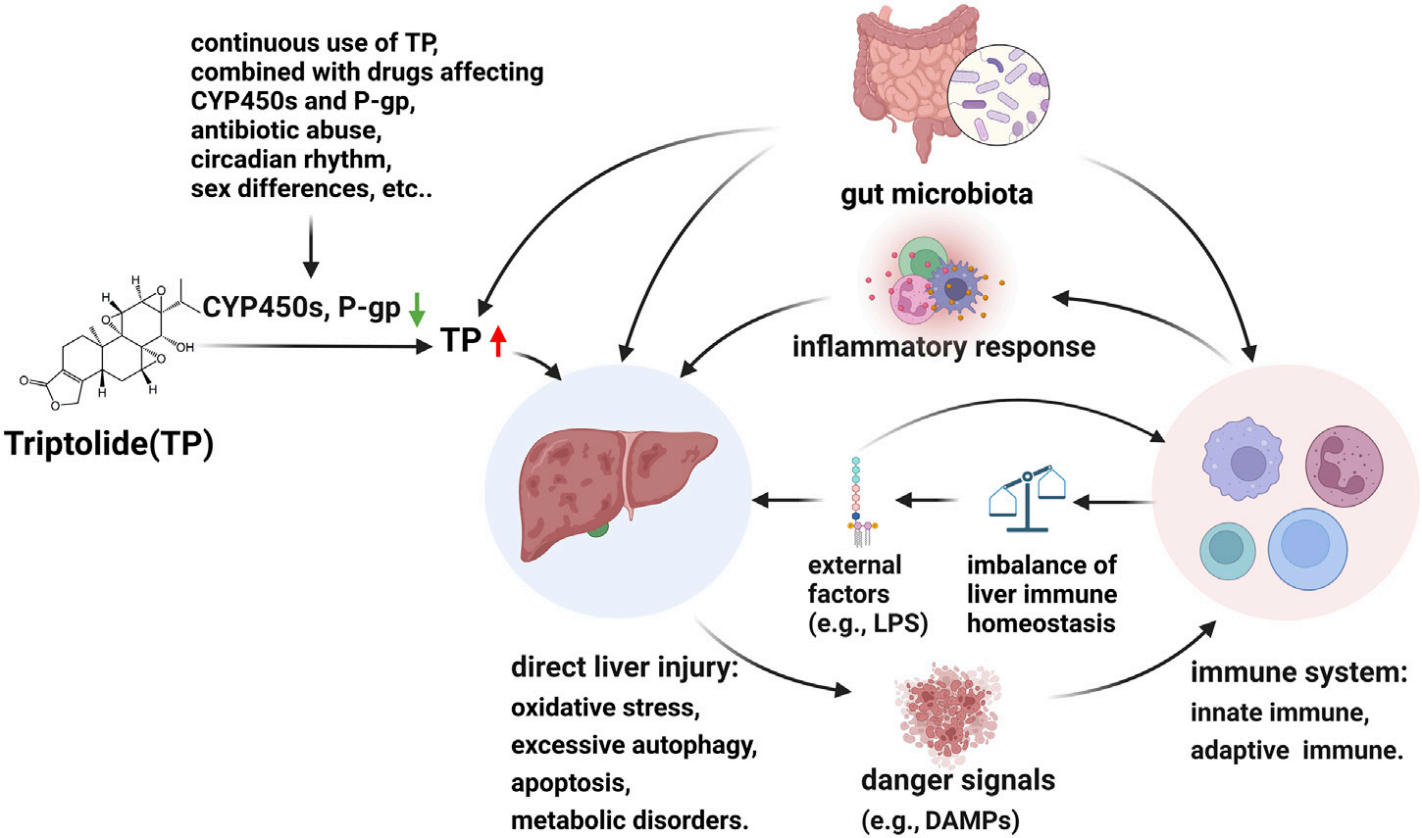
Hypothesis of molecular pathogenesis of TP-induced hepatotoxicity. CYP450 enzymes are involved in TP metabolism and P-gp is involved in TP transport. Some factors such as the continuous use of TP, combined with drugs affecting CYP450s and P-gp, antibiotic abuse, circadian rhythm, and sex differences, should affect the level of TP in the liver. TP itself is hepatotoxic and direct liver toxicity due to excessive exposure to TP, such as oxidative stress, excessive autophagy, apoptosis, metabolic disorders, etc. Cell damage caused by direct hepatotoxicity releases danger signals, such as DAMPs, which can activate the innate and adaptive immune systems, and then a large number of pro-inflammatory factors will be released. The inflammatory response further aggravates liver injury by promoting cell death, inducing excessive autophagy and metabolic disorders, etc. In addition, the imbalance of hepatic immune homeostasis caused by changes in immune cells makes the liver sensitive to external factors, such as LPS, then aggravates hepatotoxicity. Furthermore, gut microbiota mediates the hepatotoxicity of TP by affecting TP *in vivo* exposure, metabolic homeostasis, and immunity. ↓: downregulation of CYP450s and p-gp; ↑: abnormal increase TP exposure.

In this section, we propose an overall hypothesis for a possible mechanism of TP-induced hepatotoxicity. TP-induce hepatotoxicity is dose-dependently (Yuan et al., 2019a; Zhao J. et al., 2020). Therefore, hepatic CYP450s and P-gp, which affect TP exposure, play a vital role in TP-induced hepatotoxicity. Some factors such as the continuous use of TP, combined with drugs affecting CYP450s and P-gp, antibiotic abuse, circadian rhythm, and sex differences, should affect the level of TP in the liver. TP itself is hepatotoxic (Titov et al., 2011), and direct liver toxicity due to excessive exposure to TP, such as oxidative stress, excessive autophagy, apoptosis, metabolic disorders, etc. Cell damage caused by direct hepatotoxicity releases danger signals, such as DAMPs, which can activate the innate and adaptive immune systems, such as activation of NLRP3, recruitment of macrophages, Th17s, etc., and then a large number of pro-inflammatory factors will be released. The inflammatory response further aggravates liver injury by promoting cell death, inducing excessive autophagy and metabolic disorders, etc. In addition, the imbalance of hepatic immune homeostasis caused by changes in immune cells makes the liver sensitive to external factors, such as LPS, then aggravates hepatotoxicity. Furthermore, gut microbiota mediates the hepatotoxicity of TP by affecting TP *in vivo* exposure, metabolic homeostasis, and immunity (Figure 2).

Moreover, a comprehensive analysis of the toxicological mechanisms of TP could advance our understanding of the detoxification effect of TP. Increasing the expression of hepatic CYP450s and P-gp, activating Nrf2, Notch1, FXR, inhibiting excessive autophagy, regulating mitochondrial fission protein Drp1, and suppressing immune hyperactivation, are expected to become key strategies for TP detoxification. Studies show that there are variety of drugs, such as chlorogenic acid (Wang J. M. et al., 2018), and Catalpol (Zhang et al., 2022b), etc., alleviated TP-induced hepatotoxicity by modulating key targets or biological processes such as Nrf2, and autophagy (Table 1).

**TABLE 1:** Detoxification strategies for TP.

Drug	Model	Treatment	Effector Mechanisms	References
Arctiin	BALB/C mice	pretreatment with Arctiin (500 mg/kg i.g. 12), TP (0.6 mg/kg, i.g. 24 h)	Nrf2	Zhou et al. (2020)
	HepG2 cells	pretreatment with Arctiin (50 μM, 12 h), TP (50 nM, 12 h)		
Catalpol	HepaRG cells	pretreatment with Catalpol (40 μg/L 12 h), TP (20 μg/L 12 h)	inhibit excessive autophagy	Zhang et al. (2022b)
Catalpol	Female SD Rats	TP (1.2 mg/kg) + Catalpol (2.4, 24, 240 mg/kg) i.g. 14d	regulate phase I and II detoxification enzymes of TP	Fu et al. (2020)
	L-02 cells	pretreated with Catalpol (2 μg/ml, 10 μg/ml, 50 μg/ml, and 250 μg/ml 12 h). TP (20 μg/ml 36 h)		
	HepG2 cells	pretreated with Catalpol (0.4 μg/ml, 2 μg/ml, and 10 μg/ml, 12 h), TP (40 μg/ml, 36 h)		
Chlorogenic acid	Male Kunming mice	pretreatment with Chlorogenic acid (10,20 and 40 mg/kg, i.g. 7 d), TP (1 mg/kg, i.g. 24 h)	Nrf2	Wang et al. (2018a)
Epigallocatechin-3-gallate	Female C57BL/6 mice	pretreated with Epigallocatechin-3-gallate (5 mg/kg, i.g. 10d), TP (0.5 mg/kg, i.g. 22 h)	Th17/Treg balance	Yu et al. (2016)
Isoliquiritigenin	Male ICR mice	pretreatment with Isoliquiritigenin (25 and 50 mg/kg i.g. 7 d), TP (1.0 mg/kg, i.p. 24 h))	Nrf2, bile acid metabolic homeostasis	Hou et al. (2018)
	L-02 cells	pretreatment with Isoliquiritigenin (2.5,5.0, 7.5 μM 12 h); TP (50 nM, 24 h)		
Isoliquiritigenin	Male ICR mice	pretreatment with Isoliquiritigenin (50 mg/kg i.g. 7d), TP (1.0 mg/kg, i.p. 6 h), Isoliquiritigenin (50 mg/kg i.g. 18 h)	Nrf2	Cao et al. (2016)
Licorice root extract	Male Wistar rats	pretreatment with Licorice root extract (120,240 and 480 mg/kg, i.g. 7 d), TP (0.6 mg/kg, i.g. 18 h)	Nrf2	Tan et al. (2018)
	L-02 cells	pretreatment with Licorice root extract (30, 60, and 90 μg/ml, 24 h), TP (80 nM, 18 h)		
magnesium isoglycyrrhizinate	Male Wistar rats	pretreatment with magnesium isoglycyrrhizinate (13.5 mg/kg, i.g. 7 d), TP (0.6 mg/kg, i.g. 18 h)	Nrf2	Tan et al. (2018)
	L-02 cells	pretreatment with magnesium isoglycyrrhizinate (30 μg/ml, 24 h), TP (80 nM, 18 h)		
Quercetin	Female C57BL/6 mice	pretreatment Quercetin (20, 50 and 80 mg/kg i.g. 10 d), TP (0.5 mg/kg, i.g. 22 h)	Th17/Treg balance	Wei et al. (2017)
Vitamin C	Male C57/BL6 mice	pretreatment Vitamin C (250 mg/kg i.g. 12 and 24 h), TP (1.0 mg/kg, i.p. 24 h)	mitigation of oxidative stress	Xu et al. (2019)

However, research on the mechanism of TP-induced hepatotoxicity lacks depth. For example, studies have found that the role of autophagy in a model of TP-induced hepatotoxicity is controversial. It seems that proper autophagy protects TP-induced hepatotoxicity, yet excessive autophagy increases TP-induced hepatotoxicity (Wei et al., 2019; Zhang et al., 2022b). However, there is currently no clear indicator to determine the extent of autophagy in TP-induced hepatotoxicity, and it cannot tell us how to regulate autophagy. Therefore, it is necessary to develop appropriate experimental models to clarify the role of autophagy in TP-induced hepatotoxicity and look for the key indicators or biomarkers. Furthermore, multiple mechanisms have been demonstrated to be involved in TP-induced hepatotoxicity while reduce the expression of these targets does not seem to significantly alleviate TP-induced hepatotoxicity. This suggests that TP-induced hepatotoxicity is caused by the interaction of multiple factors and the key pathogenesis of TP-induced hepatotoxicity has not yet been identified. Thus, key pathogenic mechanisms need to be discovered and validated for detoxification strategies for TP-induced hepatotoxicity.
